# Predicting new crescent moon visibility applying machine learning algorithms

**DOI:** 10.1038/s41598-023-32807-x

**Published:** 2023-04-24

**Authors:** Murad Al-Rajab, Samia Loucif, Yazan Al Risheh

**Affiliations:** 1 College of Engineering, Abu Dhabi University, Abu Dhabi, UAE; 2 College of Technological Innovation, Zayed University, Abu Dhabi, UAE

**Keywords:** Astronomy and planetary science, Engineering, Computational science, Computer science, Information technology, Scientific data, Software

## Abstract

The world's population is projected to grow 32% in the coming years, and the number of Muslims is expected to grow by 70%—from 1.8 billion in 2015 to about 3 billion in 2060. Hijri is the Islamic calendar, also known as the lunar Hijri calendar, which consists of 12 lunar months, and it is tied to the Moon phases where a new crescent Moon marks the beginning of each month. Muslims use the Hijri calendar to determine important dates and religious events such as Ramadan, Haj, Muharram, etc. Till today, there is no consensus on deciding on the beginning of Ramadan month within the Muslim community. This is mainly due to the imprecise observations of the new crescent Moon in different locations. Artificial intelligence and its sub-field machine learning have shown great success in their application in several fields. In this paper, we propose the use of machine learning algorithms to help in determining the start of Ramadan month by predicting the visibility of the new crescent Moon. The results obtained from our experiments have shown very good accurate prediction and evaluation performance. The Random Forest and Support Vector Machine classifiers have provided promising results compared to other classifiers considered in this study in predicting the visibility of the new Moon.

## Introduction

Islam is considered the second religion in the world. Hijri is the Islamic calendar, also known as the lunar Hijri calendar. This is because each month starts in the Hijri calendar when the new crescent Moon is first sighted after the birth of a new Moon. Although almost all countries in the world use the Gregorian calendar for easy communication, The Hijri calendar is used by Muslim countries and Muslims, in general, across the world when it comes to religious events. Ramadan is among the holy months for Muslims. Deciding on the start of Ramadan has always been a challenging mission, and as a result, not all Muslims start Ramadan synchronously. The main reason is due to the reliance on individuals' observations of the new crescent Moon which depends on several factors that affect the final results, such as the tools used for Moon observation, sky status whether clear or cloudy, the location from where the observation is conducted, the proficiency of the observers, etc.

**Figure 1:**
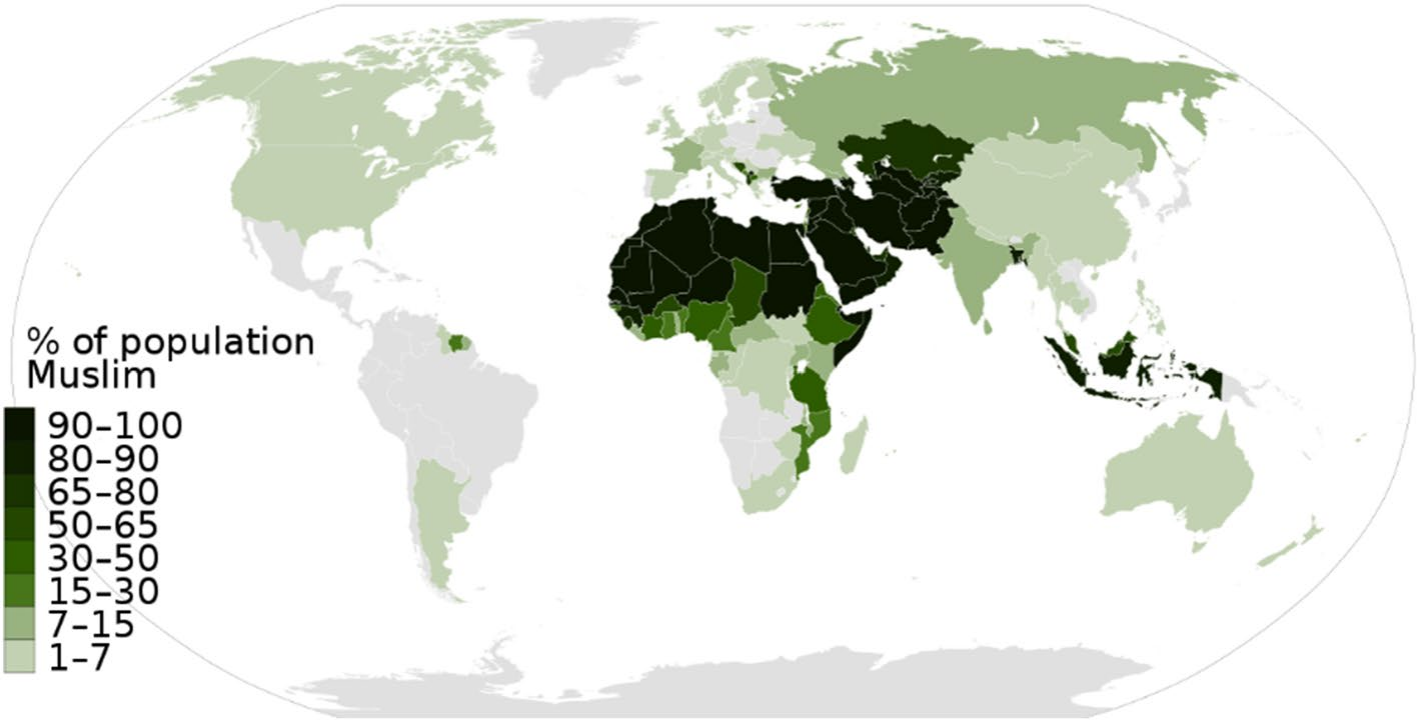
World Muslim population by percentage^3^.

Furthermore, with technological advancement, there are controversial opinions on whether to use the eye sighting of the new crescent Moon or astronomical calculation methods. The problem becomes even more serious given the number of Muslims worldwide who, each time Ramadan approaches, wait for the announcement of the first day of Ramadan. The latter is the ninth month in the Hijri calendar, and during this month, Muslim fast from before Sunrise till Sunset. Additionally, there is a need for a consensus on that day among all Muslims in the world. According to^1^ and^2^, and as indicated in Fig. 1^3^, there are about 50 countries in the world where the majority of the population is Muslim. There are approximately 30 Muslim-populated countries around the world, with more than 90% of the population of Islam. In 20 other countries, 50% to 80% of Muslim populations live in the country. In 26 countries, Islam is a national religion under the state constitution^4^.

Each year and at the end of the eighth month (Sha'ban), Muslims across the world put lots of effort and utilize different instruments such as telescopes and binoculars, astronomy, or the naked- eyes, to observe the visibility of the new crescent Moon in order to confirm the start of the holy month of Ramadan. Most of the time, it is difficult to predict the start of the month in advance, resulting in a division between Muslims fasting on different dates. The new crescent Moon has always been a matter of attention since the early decades of Islamic astronomers. Predicting the visibility of the birth of the new Moon from a given location on earth is still a challenging problem for many astronomers and mathematicians^5^. In the second half of the first century^2,6^, The priest and astronomer of Babylon developed sophisticated numerical algorithms to predict the motion of the Moon, such as the times of synodic phases and the first visibility of the new crescent Moon above the western horizon just after the Sunset^5^. Later, multiple religious scholars developed more precise methods that are now refined^5^.

All these are still not enough, and the challenge continues to exist in deciding on the start of the month of Ramadan within the Muslim community. Artificial Intelligence and machine learning, in particular, have shown their success in several fields. Machine Learning algorithms have been extensively used for classification and prediction in complicated problems such as astronomy, health sector, geology, archaeology, etc. Data regarding the visibility of the new crescent moon is complex and non-linear in nature, making it difficult to analyze with traditional methods. However, machine learning algorithms are the fitting choice in this field. They are capable of handling such data and are more efficient and accurate. By utilizing historical data and considering factors such as the time and location of the observation and weather conditions, machine learning algorithms can accurately predict future moon visibility. This is particularly important given that new observations and data are continually being collected in various regions worldwide, and the efficiency of machine learning algorithms enables timely and accurate predictions, particularly for holy months such as Ramadan, Haj, and Eid. To fill in this gap and to take full advantage of machine learning algorithms, this paper applies commonly used machine learning classification algorithms to predict the visibility of the new crescent Moon.

The remainder of the paper is organised as follows. “Background” section discusses the background and introduces important terms used in this study. “Literature review” section reviews previous research studies. "Experiment methodology" section illustrates the experiment methodology. “Experimentation” section presents the experimentation with the dataset built and the software used in the experimentation. “Results and discussion” section discusses the obtained results. Finally, “Conclusion and future work” section concludes this paper.

## Background

The Gregorian calendar, which is the dominating one used worldwide, was introduced in 1582 by the Pope Gregory XIII^7^. Although Islamic states and Muslims over the world utilize the Gregorian calendar for easy communication and coordination purposes, they use the Hijri calendar to spot important dates and religious events.

**Figure 2:**
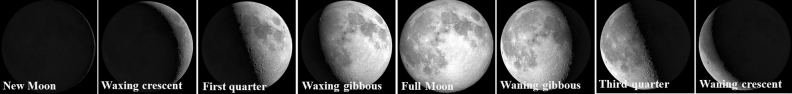
Moon phases.

The Hijri calendar is a lunar calendar and consists of twelve months but, in total about eleven days shorter than the Gregorian calendar. Ramadan is the ninth month in the Hijri calendar and is considered among the holly months for Muslims. The start of each month of the Hijri calendar is marked by the sighting of a new crescent Moon. The Moon constantly moves in relation to the earth and the Sun. By doing so and depending on its position relative to the Sun, a part of it is lit up (i.e., a fraction of the Moon illuminated, known as Moon illumination), resulting in changing its appearance, also known as a phase. Therefore, the Moon goes through eight different phases^8^, starting from the new Moon, which is invisible, then a very thin crescent lit up by the Sunlight and called the new crescent Moon, as shown in the first picture of Fig. 2, followed by the waxing crescent, and moves to the next phase till it reaches the last phase then back starting a new cycle, as depicted in Fig. 2, taken from NASA website^8^.

**Figure 3:**
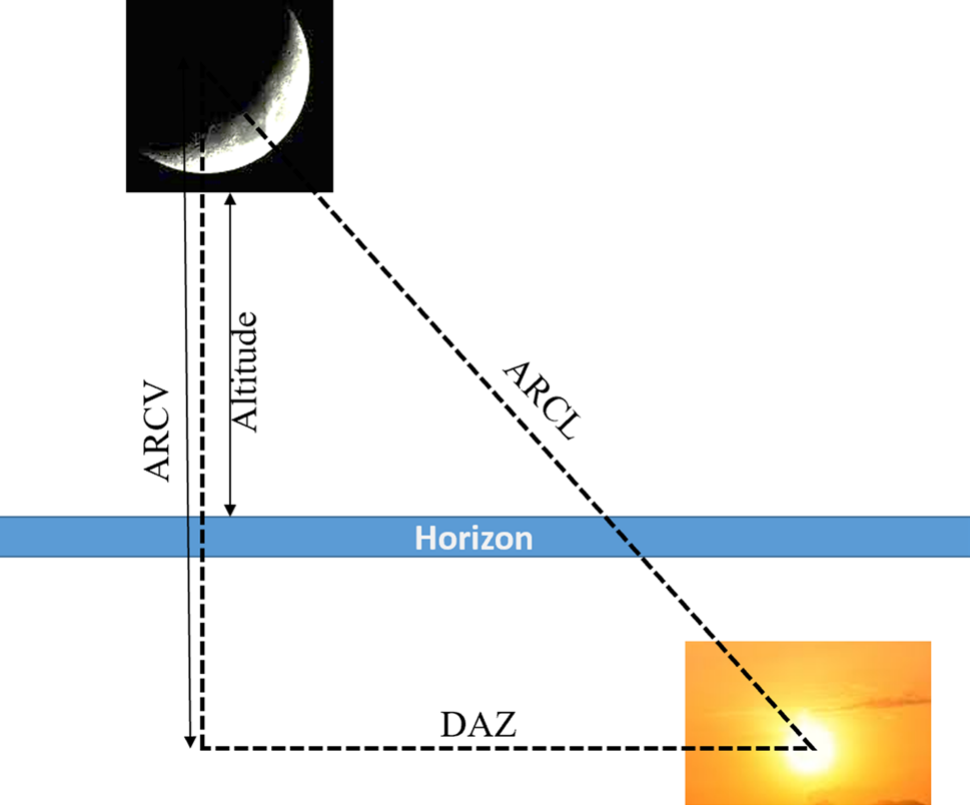
The relative positions of the Moon and Sun and parameters used in the crescent Moon visibility prediction.

**Figure 4:**
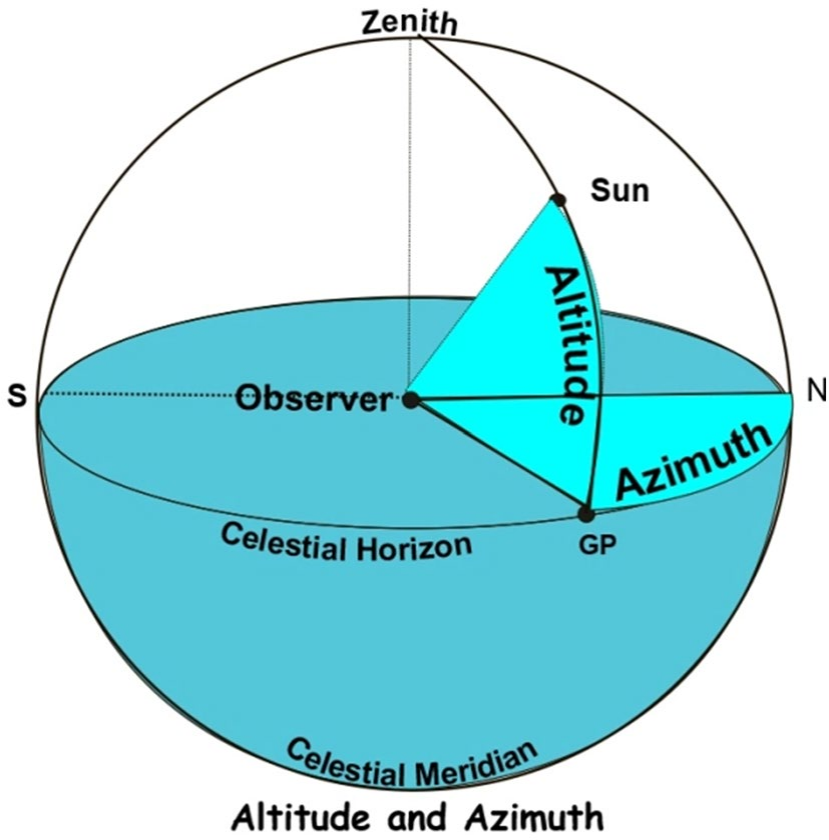
Altitude and Azimuth^13^.

The visibility of the new crescent Moon has always been a debate since the Babylonian age. Scientists and astronomers (Muslim and non-Muslim), and scholars had contributed to this by suggesting parameters and criteria to determine the visibility of the new crescent Moon. To name a few are Ibn Tariq, Al- Khawarizmi, Al-Tabari, Al-Biruni, Ibn Sina, Futhringham^9^, Maunder^9^, and Bruin^10–12^. As mentioned earlier, the visibility of the new crescent Moon is essential as it marks the beginning of a month and helps in setting up the Hijri calendar and determining religious events. Before reviewing the parameters and criteria considered by researchers, let us first introduce some terminology that will be used later in the next sections. We refer to Figs. 3 and 4^13^, which show the Moon and the Sunset. Among the important parameters that are considered in this study are:

- The Moon's altitude is the height of the Moon above the horizon.
- The ARCV is the arc of vision at Sunset time.
- ARCL, the ARC of the Light, also known as elongation, is defined as the angular distance between the Moon and the Sun^13^. ARCL is also used to calculate the width of the crescent Moon, W.
- The Azimuth' of an object defines its position on the celestial sphere in the horizontal coordinate system, measured in degrees starting from 0 north and turning in a clockwise direction^14^, as shown in Fig. 4.
- DAZ, Difference in Azimuth, is another parameter that gives information about the Moon/Sun position. DAZ is defined as the angular difference in azimuth between the Sun and the Moon, as shown in Fig. 3.
- The Moon conjunction is the birth of a new Moon, and it occurs when the Moon passes between the earth, and the Sun in which case the part lit up by the Sun is facing the Sun, and therefore the Moon is not visible to us.
- The Moon's lag time is the difference in time between the Sunset and the Moonset.
- The Moon's age is defined as the elapsed time from the birth of the new Moon till the Sunset on the day of observation.

As discussed, parameters and criteria putting threshold values to these parameters to validate the visibility of the new crescent Moon were proposed by scientists, astronomers, and scholars. In Section "Literature review, we will cover these criteria in more detail.

## Literature review

In this section, we present our methodology followed in conducting a systematic literature review, and then we will discuss the results found in the related works.

### Literature review searching methodology

The title Moon phase observation using machine learning is not frequent. So, we have conducted our search for all types of papers and for all years without exception. We have searched several digital resources and databases, such as IEEE Xplore, Science Direct, Scopus, Springer Link, and Google Scholar, for related articles. Several keywords were used for the searching process such as ('the' and 'month' and 'of' and 'Ramadan'), ('Hijri' and 'calendar'), ('Moon' and 'phases'), ('machine' and 'learning'), ('Moon' or 'observation'), ('lunar' or 'crescent'), ('lunar' or 'visibility'), ('crescent' or 'visibility'), and ('machine' and 'learning' and 'for' and 'Moon birth' or 'visibility').

**Figure 5:**
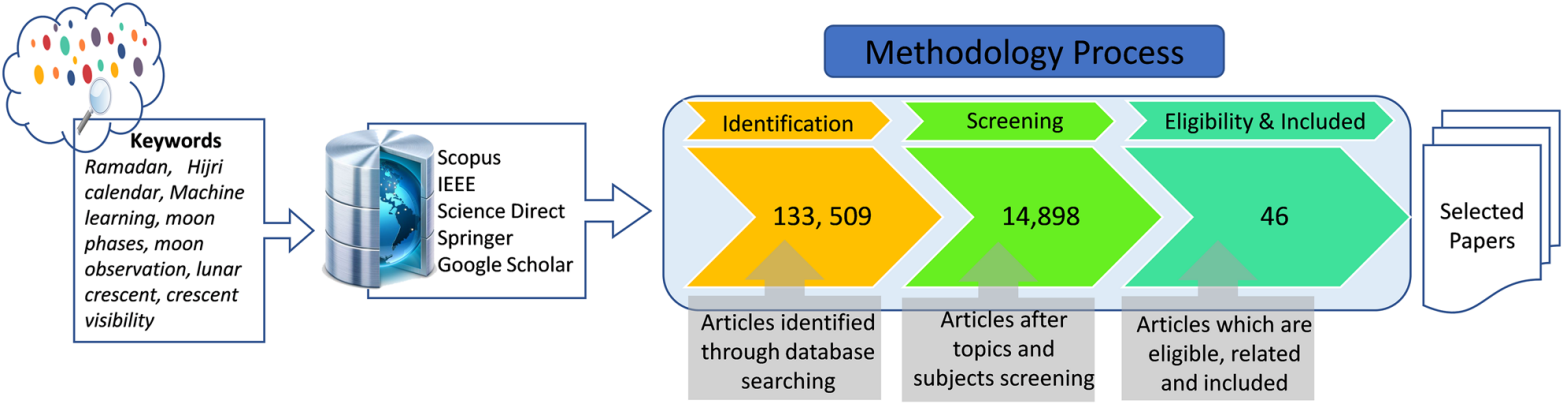
Literature review searching process.

Figure 5 shows the process followed in selecting the related articles found in our search. A total of 133,509 articles were identified. Then, a screening process was conducted to exclude articles not really relevant to our study, and we have selected only articles which are related to computer science, astronomy, machine learning, artificial intelligence, and data analysis. We ended up with 14,898 articles, which have been passed on a second screening process after screening topics and subjects. After careful reading of these articles, and therefore, only 46 papers have been identified and summarized in the related work sub-section.

### Related work

As stated earlier, many societies depend on the lunar phase cycle for their calendar The establishment of the lunar calendar is based on the observation of the first sighting of the new crescent Moon. Most Islamic countries and Muslims across the world depend on the Islamic calendar converter, which is based on the arithmetical (or tabular) calendar to predict the approximate beginning of the new month. This arithmetical calendar was introduced earlier by Muslim astronomers during the ninth century. However, this predicated calendar has an error of 4 × 10^–4^ days per 30 years, which accumulates to a whole day in 2492 years^15^.

It is obvious that in order for the new crescent Moon to be sighted, a minimum brightness contrast between the Moon and the sky is required. Starting with the criteria set by the Babylonians, who stated that the visibility of the new crescent Moon with naked-eyes at local Sunset was subject to two conditions: the age of the Moon must be more than 24 h and the Moon's lag time must be greater than 24 min^11^. Al-Khwarizmi's criterion for the new crescent Moon to be sighted was the arc of separation of the Sun and Moon along the celestial equator must be greater than 12°. On the other hand, Danjon's criterion^16^ for the new crescent Moon visibility with the naked-eyes was that the ARCL must be at least of 7°. Al-Battani^17^ suggested the use of the parameters DAZ, Moon altitude, and the width of the Moon, W, for the visibility of the new crescent Moon. Ilyas^18^ set the condition for the new crescent Moon to be sighted is that the ARCL must be at least in the range of 9° to 10°. McNally^19^ argued Danjon's work stating that the new crescent Moon shortening is mainly due to atmospheric conditions. In another study^20^, Ilyas updated the condition of the new crescent Moon visibility for the ARCV to be 10.5°. According to the authors in^21^, the visibility of the new crescent Moon is unpredictable using only one of the above-mentioned parameters. For example, with only the Moon Age or Lag, there is no predictive value. In order to correctly calculate the Moon's appearance, several astronomical conditions are combined to calculate the Moon's appearance, such as the minimum altitude of the new crescent Moon above the horizon, the minimum ARCL, and the minimum age of the Moon since the conjunction occurrence.

Doggett et al.^22^ reported the new crescent Moon observation results of five monophases throughout North America. They found that ancient and medieval observation rules are highly unreliable. They also claimed that recent empirical criteria such as the relative altitude and azimuth of the Moon at Sunset time would reflect a very good observation accuracy. They claimed that information about atmospheric, optical, and human factors would have an effect on observations. Yallop^23^ examined Maunder^9^, Indian Astronomical Ephemeris and Bruin^10^ methods. The first two methods considered the parameters DAZ and ARCV, while the last method considered ARCV as a function of Yallop came up with the formulae of the time of the best visibility of the new crescent Moon. Also, Schaefer^24^ and based on over 200 observations, suggested that the atmospheric conditions must be considered in the new crescent Moon visibility process. He argued that using the empirical method cannot be applied in all situations, and this is because it depends on the observer's location and other factors. For that, Schaefer developed an algorithm to predict the visibility of the new crescent Moon. The program takes as input the set of observing conditions such as the date of the new Moon, observer location in terms of latitude and longitude, observer age, atmospheric conditions, and the program generates the age of the Moon at the time of the first visibility for the given conditions. Fatoohi et al.^25^, on the other hand, discussed all the work suggested by Danjon, Illyas, McNally, and Schaefer and concluded that the minimum value of the ARCL, which is 7.5°, is a reasonable value for the new crescent Moon to be visible.

In 1998, the Islamic Crescent Observation Project (ICOP for short), an initiative of the Arab union of astronomers and space sciences and the Jordanian astronomy society^11^, was established to serve as a central point for gathering information about the observation of the new crescent Moon at the beginning of each month and this from different countries across the world and creating a central database for that. Odeh^11^ claimed that the accurate prediction of the visibility of the crescent Moon could not be achieved by a single parameter. Furthermore, he added that the Moon's age is not a good indicator of its brightness. Odeh proposed a new criterion for the prediction of the visibility of the crescent Moon, which is the W (in arc minutes), and which must be used in conjunction with the ARCV, yielding the equation of visibility prediction of the new crescent Moon, Odeh suggested four possible scenarios based on the value of V to decide whether the new crescent Moon is visible with naked-eyes, by optical aid and can be seen by naked-eyes, visible only by optical aid, not visible at all. The other important Odeh contribution was the creation of a database of lunar crescent observations from different sources as part of the ICOP project to come up with a total of 737 observations. Caldwell^26^ studied the effects of the Moon's lag time and the ARCL on the visibility of the new crescent Moon with naked-eyes or binoculars. He conducted a simulation and derived equation setting boundaries where visibility criterion lines were suggested. He derived the values of the Moon's lag time as a function of the ARCL to find out the three lines to confirm the visibility of the new crescent Moon, whether with naked-eyes or binoculars or the new crescent Moon visibility is impossible. Özlem's work^27^ aimed to propose a visibility criterion, which can also be used for thicker crescents, including daytime visibility, when the Moon is visible together with the Sun.

Alrefay et al.^28^ claimed that predicting the visibility of the new crescent Moon is a challenging and difficult process for several technical reasons. The lunar calendar cannot be calculated on the basis of observations since the start of the next month requires about 29 days of waiting. Such observation calendars are highly influenced by atmospheric conditions and usually involve errors caused by human factors such as illusions. The authors in this study applied several criteria from 545 observations over 27 years (1988–2015) from different locations in Saudi Arabia. They developed a new criterion based on the lunar W and the ARCV. According to the 1978 Turkish International Conference, the majority of Muslim countries agreed that the Islamic month begins when the ARCL is greater than 8° and the new Moon height from the horizon when the Sunset must be greater than 5°^29,30^.

Some researchers suggested systems or applications to help in the detection of the first crescent Moon or the computation of the Hijri calendar. For instance, Alhammadi et al.^31^ proposed an electronic system that uses inputs from a database; the inputs suggest the location to which the system camera should be oriented. This location is based on the DAZ and the Moon's latitude. Then, the images captured by the camera are processed to confirm whether these images correspond to new crescent Moon or not. A desktop application was proposed in^32^ to compute Hijri calendar dates and displays all Islamic events in the year.

Recently, the advent of computational methods and the availability of the immense data generated from satellites in orbit spaces have changed the way of analyzing and processing the data. For example, NASA's Space Program provides us with massive data on cosmic objects to help us exploring space. Astronomy experts have examined images generated and collected by telescopes like Hubble. Later, with the introduction of advanced telescope technologies and satellites such as Kepler, these have opened new horizons to autorotate the analysis process of observation and the need to learn the computer machine to do the tasks. These satellites and telescopes process data using various features and imaging techniques, not only to capture images but also to identify these exoplanets^33,34^. In their paper, Moshayedi et al.^35^ have presented a prototype system that utilizes machine learning models to detect the various phases of the moon. The primary focus of their research is centered on analyzing a collection of moon images to determine the lunar phase that corresponds to each image. It is worth noting that their approach differs from our own work, as our investigation is based on a dataset consisting of numerical data that pertains to distinct moon phases, rather than image analysis. Our specific aim was to identify the appearance of the new crescent moon, which serves as the marker for the commencement of the Hijri month. The authors in^36^ proposed an image processing-based method to detect the crescent Moon from observed images. The processing went through several phases like a Gaussian smoothing filter to remove noise, followed by the quality enhancement of the image focusing on the part containing the crescent Moon. The authors used the circular Hough transform to extract the crescent Moon from the background of an image and detect the center of the Moon and the shape of the crescent. In the same context, Sejzei and Jamzad^37^ developed a toolbox in Matlab that enhances the image of a crescent Moon and helps observers to see/detect the crescent Moon.

Artificial intelligent techniques and algorithms such as Machine (ML) and Deep Learning (DL) algorithms represent powerful tools in processing the immense data of extrasolar planets. More recently, researchers have suggested the use of ML algorithms for the classification of astronomical objects^38–43^. Some of these common algorithms which were applied to classify objects found in the Kepler Cumulative Object of Interest are Random Forest (RF), Support Vector Machines (SVM), AdaBoost, and Deep Neural Networks (DNN)^33,41^. Beniwal et al.^40^ applied classical ML algorithms for pulsar classification.

Tafseer^44^ considered the problem of Moon crescent visibility for each month as a classification problem and suggested the use of ML algorithms instead of mathematical or astronomical methods. His study was based on the dataset found on the website managed by ICOP. A pre-process of the dataset was conducted where 1070 samples with cloudy or partially cloudy sky condition were removed. The author added some astronomical features such as the age of the Moon, the Moon's lag time, the altitude difference, DAZ, the Moon phase, and the atmosphere. Four algorithms were considered in this study, namely logistic regression, Neural Network, SVM, and RF, applied to 1522 samples, among which 80% were used for the training phase and 20% were used for the testing phase. Results obtained in this study showed that RF achieved better precision in predicting the visibility of the crescent Moon with 88%. In another study^45^, the author employed an Artificial Neural Network (ANN) model to forecast the visibility of the new crescent moon. However, the research was limited to the use of a single machine learning algorithm (ANN) and was restricted to a singular geographical location, specifically Iraq.

The unification of Islamic Hijri calendar, and the accuracy of the beginning of each Hijri month, has always been a concern for the Muslim community all over the world. The authors in^12,46^ raised the discrepancies with the beginning of the holly months in different parts of the world. Khan^46^ claimed that the only workable solutions is using astronomical calculations to reduce errors. Zainon et al. in^12^, on the other hand, discussed the criteria and factors to take into consideration in the determination of the beginning of each Islamic Hijri month, such as the conjunction, and the new crescent Moon visibility. They also suggested some required parameters for the Moon to be possibly sighted by the naked-eyes or using a sophisticated tool like telescopes, which are Moon age, Moon's lag time, Moon's altitude, ARCL, ARCV, DAZ, and W. These parameters^12,28^ are presented in Fig. 3.

In an effort to help in this matter, this current study suggests the application of ML algorithms in the prediction of the visibility of the new crescent Moon. To the best of our knowledge, in the context of this research, only^44^ and^45^ tackled the prediction of the crescent Moon visibility applying some ML algorithms. Nevertheless, Allawi^45^ was confined to a single ML algorithm, and it was just restricted to one individual country, Iraq. Referring to the dataset used in^44^, several limitations were identified such as DAZ feature which should be positive numbers, the values of some features like ARCL (elongation) were not accurate, names of some countries and cities features were not consistent; same countries and cities in different languages or abbreviated, which may affect the analysis results. To overcome all these shortcomings, we built our own dataset and included all the years up to the current year 2022, as explained in "The proposed model" section.

## Experiment methodology

The main contribution to this research is to figure out the best machine learning classifier that can assist in predicting the eye visibility observation of the new crescent birth and specially the start of the holy month of Ramadan.

### The proposed model

**Figure 6:**
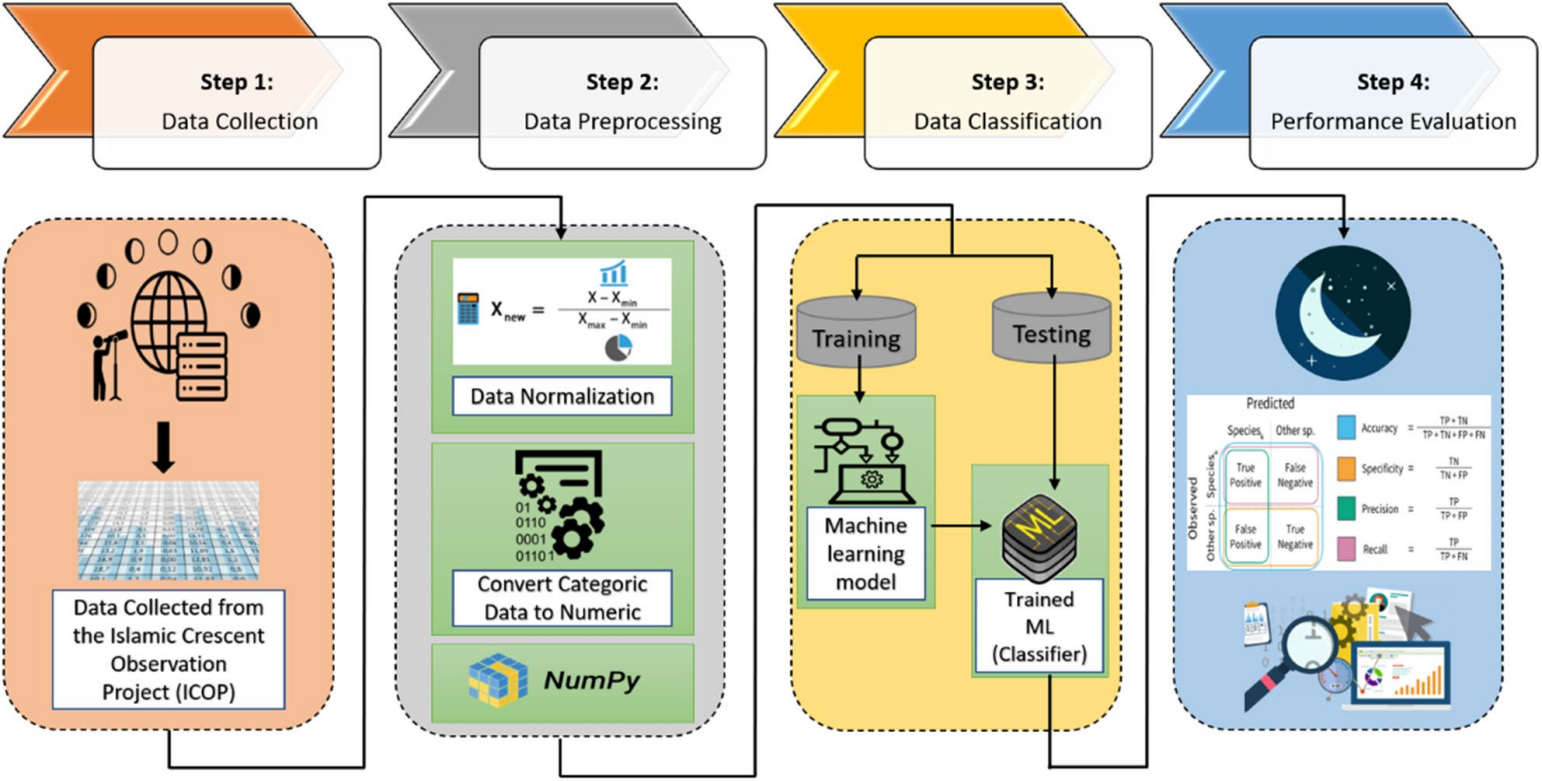
Steps followed in our analysis.

In this paper we collect the data, filter and pre-process the data, and apply ML for the process of predicating the new crescent moon birth. Figure 6 illustrates the steps of how the experiment of this paper is conducted in terms of collecting the data and applying the machine learning algorithms. The process involves the following steps.

**Figure 7:**
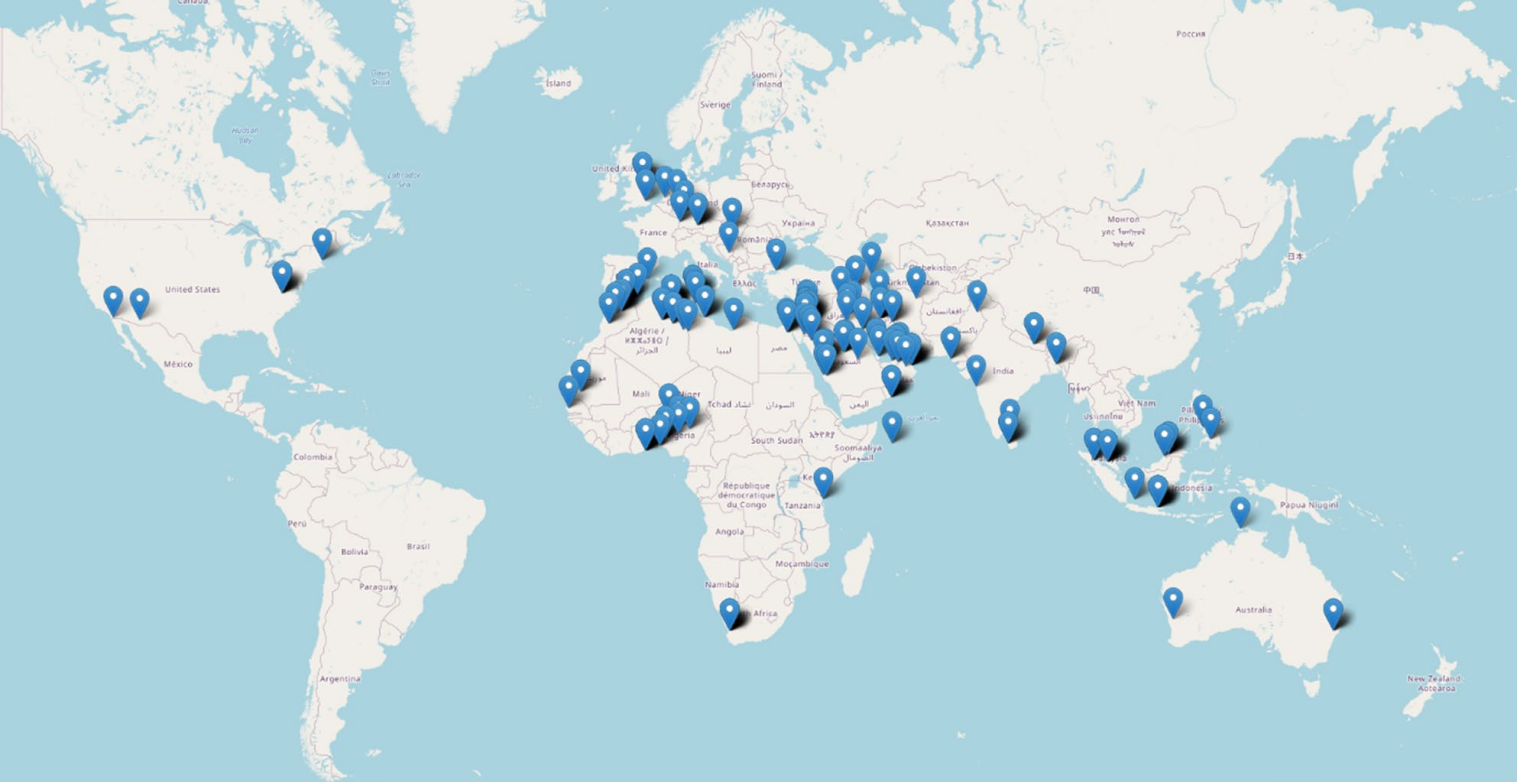
Locations of the observations for the 13 years. Python 3.6.9, Collab URL: https://colab.research.google.com/.

*Step 1—Data collection* To commence our study, we began by collecting data to construct our dataset. We ensured that our dataset was thorough and adhered to the necessary standards by sourcing data from the International Crescent Observation Project (ICOP). Our data collection involved recording observations and all associated features of multiple states and cities in each country registered by the ICOP. The data was accumulated over a period of 11 years from 2009 (1431 Hijri) to 2020 (1441 Hijri). In selecting the features to include in our dataset, we utilized common features used in this type of study while also adding our own features, such as ARCL and illumination (previously mentioned, aiding in calculating W). Other features encompassed in our dataset are date, country, city, state, atmosphere, latitude, longitude, Sunset, Moonset, Sun_Moon_Lag, Age_of_Moon, MoonAltitude, SunAltitude, altitude_Difference, MoonAzimuth, SunAzimuth, azimuth_Difference (DAZ), and V_eye. Table 1 outlines the important formulas for feature calculations. To focus solely on data relevant to the Ramadan month, we removed all the Hijri months except for Ramadan. Additionally, we added data pertaining to the last two Hijri years (1442 and 1443), including the current year 2022, by utilizing Accurate Times 5.6 software^47^. Our final dataset consists of a total of 358 observations from 43 countries, as depicted in Fig. 7.

**Table 1:** Formulas for some features values computation^14,26,27,44^.

Feature	Formula
Age_of _Moon	$sunset-conjunction$
Sun_Moon_Lag	$Moonset-Sunset$
altitude_Difference	$MoonAltitude-SunAltitude$(in rad)
Atmosphere	Superb = 1, clear = 0.85, hazy = 0.6, Very hazy = 0.5
DAZ (as function of ARCL and ARCV)	$cos(ARCL) = cos(ARCV) \times cos(DAZ)$
Illumination	½ × [1 − cos (*ARCL*)]
W	11,950 × *Illumination/Moon’s Altitude*

*Step 2—Data pre-processing* The collected data underwent pre-processing to ensure its suitability for analysis. This involved a cleaning process to remove any null or missing data, which could potentially impact the accuracy of the results. Next, the data was standardized using min_max normalization, which scales the data to values between 0 and 1, applied to some features such as atmosphere, longitude, and Sun_Moon_Lag. This normalization technique is commonly used to ensure that the data is on the same scale and facilitates the comparison of different variables during the analysis process. Before conducting our experiments, we have pre-processed our data as follows:

- All observations with missing feature values were dropped.
- We removed all rows (observations) where the Sun_Moon_Lag was found negative.
- Some countries in the dataset were in Arabic. So, all have been translated into English. Also, countries and cities that had different spellings on the ICOP website have been rewritten to meet the spelling used by the Accurate Times 5.6 software (such as Mecca to Makkah). Abbreviations have been given full names as well.
- The atmosphere feature was converted to numeric values, as shown in Table 1.
- The observation feature, V_eye, has been converted from categorical to numeric so that the machine learning model can understand, fit, evaluate, and extract valuable information. This feature is used as the label for the model.

**Figure 8:**
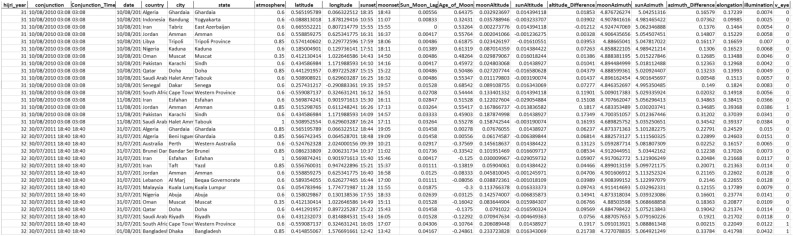
A sample of the observation features included in our dataset.

As a result, a sample of the observation features of our dataset is shown in Fig. 8.

**Figure 9:**
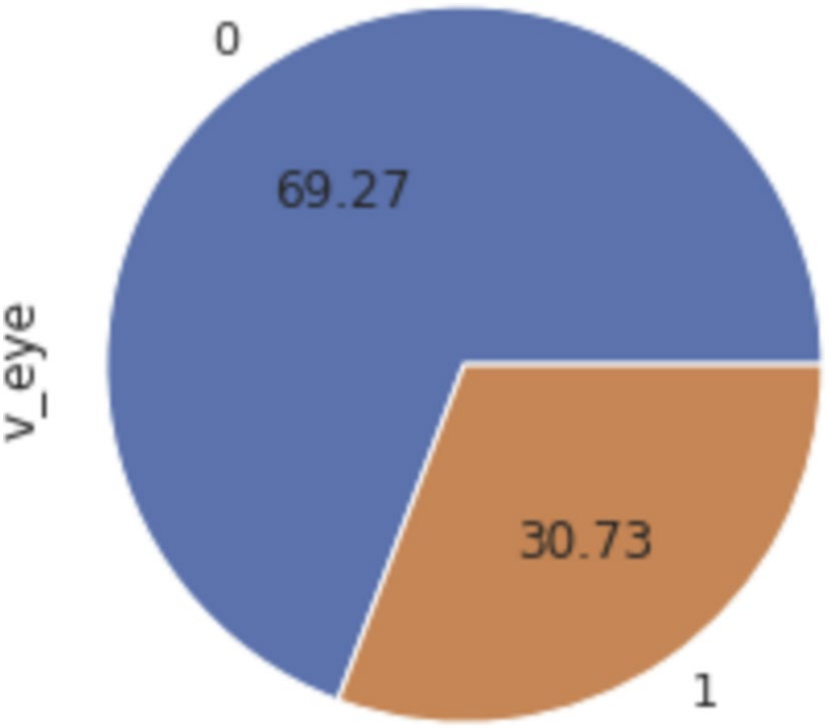
Percentage of samples of the two classes in the dataset (True = 1) and (False = 0).

It is worth mentioning that the dataset that we built was imbalanced, Fig. 9. Out of 358 observations, 248 were False observations, meaning that the new crescent Moon cannot be observed with the naked eye, while 110 were True observations. To deal with imbalanced data, there exist several techniques. One of them is to consider the Area under the Curve when evaluating the classification accuracy. We will see this in more detail in the “Results and discussion” section.

*Step 3—Data prediction using ML* A variety of common ML algorithms were trained and tested to predict the eye visibility of the birth of the new crescent Moon for the Holy month of Ramadan. The ML algorithms used in this analysis are brefly described here:

The ***K-nearest neighbors*** Classifier algorithm is a classification technique used in supervised machine learning. It is implemented in the sklearn.neighbors.KNeighborsClassifier library in Python. The algorithm works by calculating the distance between the test data point and all the training data points, using a specified distance metric such as Euclidean distance. The algorithm then selects the k-nearest neighbors (i.e., the k training data points with the shortest distances) and assigns the class label that is most common among these neighbors to the test data point.

The ***Support Vector Classification*** algorithm is a popular supervised machine learning algorithm used for classification tasks. It is implemented in the sklearn.svm.SVC library in Python. SVC works by finding a hyperplane in a high-dimensional space that maximally separates the different classes of data points. This hyperplane is defined by a subset of the training data points, known as support vectors. The distance between the hyperplane and the support vectors is maximized, resulting in a robust classification boundary.

The ***Decision Tree*** algorithm is a supervised learning algorithm used for classification and regression problems. It is based on the idea of recursively partitioning the feature space into smaller regions, such that the data in each region belongs to the same class or has similar values for the target variable. The Decision Tree Classifier implementation in scikit-learn is a binary classification algorithm that builds a binary decision tree from the training data. Each node in the tree represents a binary decision based on a feature and a threshold value. The goal of the algorithm is to recursively split the feature space into regions that are as homogeneous as possible with respect to the target variable.

The ***Random Forest Classifier*** is an ensemble learning algorithm used for classification problems. It combines multiple decision trees into a single model to improve accuracy and reduce overfitting. In scikit-learn, the Random Forest Classifier implementation builds a forest of decision trees from the training data. Each tree is constructed using a random subset of the features and a random subset of the training data. The output of the algorithm is the average prediction of all the trees in the forest.

The ***MLPClassifier*** is a supervised learning algorithm used for classification problems. It is based on artificial neural networks that are inspired by the structure and function of the human brain. MLP stands for Multi-Layer Perceptron, which is a type of neural network that has one or more hidden layers between the input and output layers.In scikit-learn, the MLPClassifier implementation uses backpropagation to train the neural network. Backpropagation is an algorithm that adjusts the weights of the connections between neurons in the network to minimize the error between the predicted and actual outputs. The alpha parameter controls the regularization strength to prevent overfitting, and the max_iter parameter controls the maximum number of iterations for the solver to converge. The neural network consists of an input layer, one or more hidden layers, and an output layer. Each layer contains a set of neurons that are connected to the neurons in the adjacent layers. The input layer receives the input features, while the output layer produces the predicted class labels. During training, the neural network learns to map the input features to the corresponding class labels by adjusting the weights of the connections between the neurons. The activation function is used to introduce non-linearity in the network and help it learn complex patterns in the data.

The ***AdaBoost Classifier*** is an ensemble learning algorithm used for classification problems. It is based on the idea of combining multiple weak classifiers into a strong classifier. In scikit-learn, the AdaBoostClassifier implementation combines decision trees as the weak classifiers to form the ensemble. The algorithm works by iteratively training weak classifiers on the data and adjusting their weights to give more emphasis to misclassified data points. The n_estimators parameter controls the maximum number of weak classifiers to be used in the ensemble. During training, the algorithm assigns initial weights to each data point in the training set. The first weak classifier is trained on the data using these weights. The algorithm then increases the weights of the misclassified data points and decreases the weights of the correctly classified data points. This process is repeated for the subsequent weak classifiers. The final strong classifier is obtained by combining the weak classifiers according to their individual performance and weights. The output of the algorithm is the class label predicted by the strong classifier.

The ***Naïve Bayes*** algorithm is a probabilistic machine learning algorithm used for classification problems. It is based on the Bayes' theorem of probability theory. In scikit-learn, the GaussianNB implementation assumes that the input data follows a Gaussian or normal distribution. The algorithm works by estimating the class conditional probability density function (PDF) of the input features for each class using the training data. The PDF is a function that describes the likelihood of observing a certain value of the input feature given a class label. During training, the algorithm calculates the prior probability of each class, which is the proportion of the training data points that belong to each class. It then estimates the class conditional PDFs of the input features for each class using the training data. During prediction, the algorithm calculates the posterior probability of each class given the input features using Bayes' theorem. The posterior probability is the probability of the input features belonging to a certain class given the prior probability and class conditional PDFs. The class with the highest posterior probability is then predicted as the output.

The ***Logistic Regression*** algorithm is a statistical machine learning algorithm used for binary classification problems. It is based on the logistic function or sigmoid function that maps any real-valued number to a value between 0 and 1. In scikit-learn, the LogisticRegression implementation uses a regularization term to avoid overfitting. During training, the algorithm estimates the coefficients or weights of the input features that maximize the likelihood of observing the training data given the coefficients. The coefficients represent the strength and direction of the relationship between each input feature and the output label. The regularization term controls the complexity of the model by adding a penalty term to the loss function that measures the deviation of the estimated coefficients from the actual coefficients. The solver parameter specifies the algorithm used to solve the optimization problem, and the random_state parameter ensures reproducibility of the results. During prediction, the algorithm calculates the probability of the output label being 1 given the input features using the logistic function and the estimated coefficients. If the probability is greater than or equal to a threshold, typically 0.5, the output label is predicted as 1, otherwise it is predicted as 0.

The corresponding parameters of the above mentioned ML algorithms are summarised in Table 2. In our research, we utilized the default ML algorithms available in the scikit-learn library, which is a widely used open-source toolkit for ML in Python. Our predictive model was developed using these algorithms, and the results obtained from our experiments showed their effectiveness. The implementation details are found in "Implementation details" section.

**Table 2:** Parameters used in the ML algorithms implemented in the study.

ML algorithms	Hyperparameters	Defined parameters
K-nearest neighbours (KNN)	n_neighbors	5
Support vector machine (SVM)	C	1.0
	Kernel type	rbf
	Gamma	Scale
Decision tree (DT)	max_depth	5
	Criterion	Gini
Random forest (RF)	n_estimators	100
	max_depth	5
	min_samples_split	2
Neural networks (NN)	Alpha	1
	max_iter	1000
	hidden_layer_sizes	100
	Shuffle	True
AdaBoost	n_estimators	100
Naïve Bayes (NB)	None	None
Logistic regression (LR)	Solver	Liblinear
	random_state	0

*Step 4* Finally, all output results were evaluated using multiple performance metrics such as the accuracy, precision, recall, F1 score, and the area under the curve to measure the performance of the machine learning algorithms.

## Experimentation

### Implementation details

To implement our models and train and test our data, we have used the Collaboratory notebook (Colab)^48^, a Google research product, which is a free and lightweight tool since it runs on the cloud. The code has been written in Python, and several python libraries have been used, such as Panadas, Numpy, Scikit-learn, Matplotlib, Seaborn, and others. Pandas has been used for data manipulation and analysis. Numpy has been used for computation, while Matplotlib and Seaborn have been used for data visualization. scikit-learn library, on the other hand, has been used for the implementation of machine learning algorithms. The algorithms considered in this analysis are those provided by the Scikit-learn library with their default parameters.

### Experiment setup

To conduct our experiments, we have used the machine learning algorithms commonly used in the literature for classification purposes, which are: Support Vector Machine (SVM), Decision Tree, AdaBoost, Random Forest, Logistic regression, Naïve Bayes, K-Nearest Neighbor, and Neural Networks. The dataset was split into 70% for training the model and 30% for the testing and validation; this splitting is commonly applied to validate the data and to assure that the classification results will not be overfitted. The selection of training and testing observations were made randomly.

**Figure 10:**
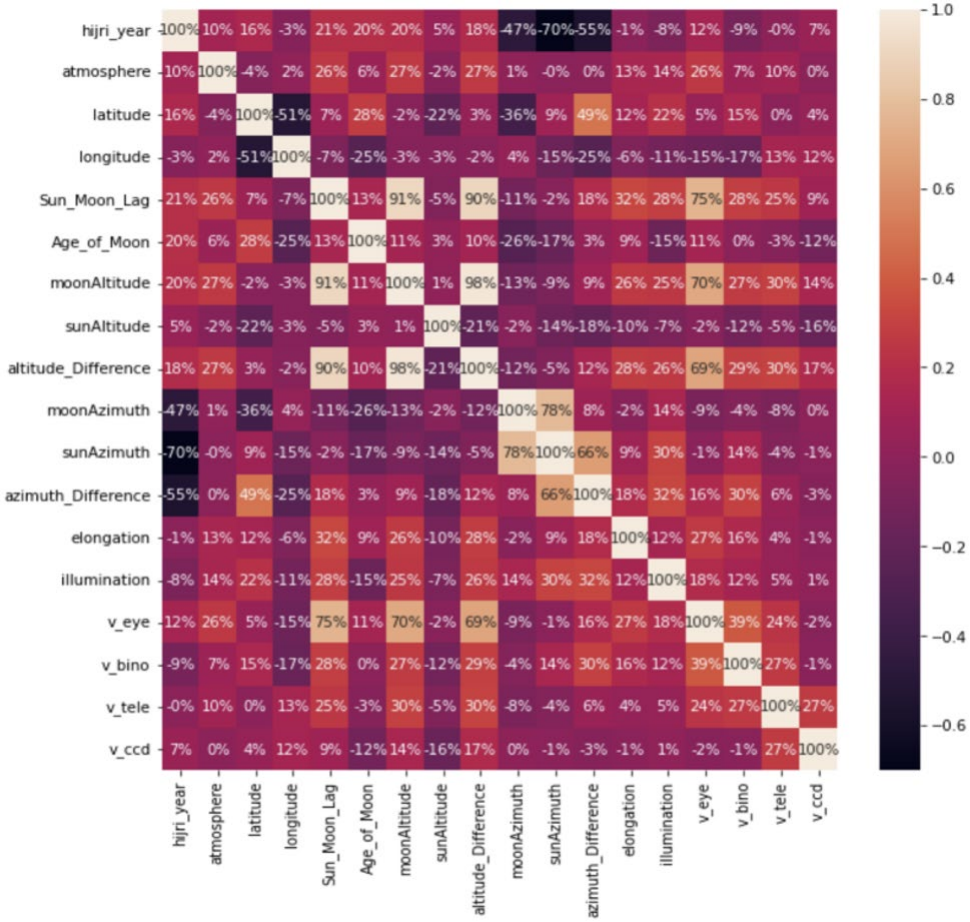
The heatmap showing correlation between features of the dataset. Python 3.6.9, Collab URL: https://colab.research.google.com/.

In the first step in the experiment, we computed the correlation between the features and obtained the following heatmap, shown in Fig. 10.

From the heatmap, we can notice that the Sun_Moon_Lag has the highest correlation of 75%, the Moon_Altitude of 70%, and the Altitude_Difference of 69%, and the other features atmosphere, Age_of_Moon, azimuth_Difference (DAZ), Elongatopn (ARCL), and illumination also have a relatively high correlation.

## Results and discussion

**Figure 11:**
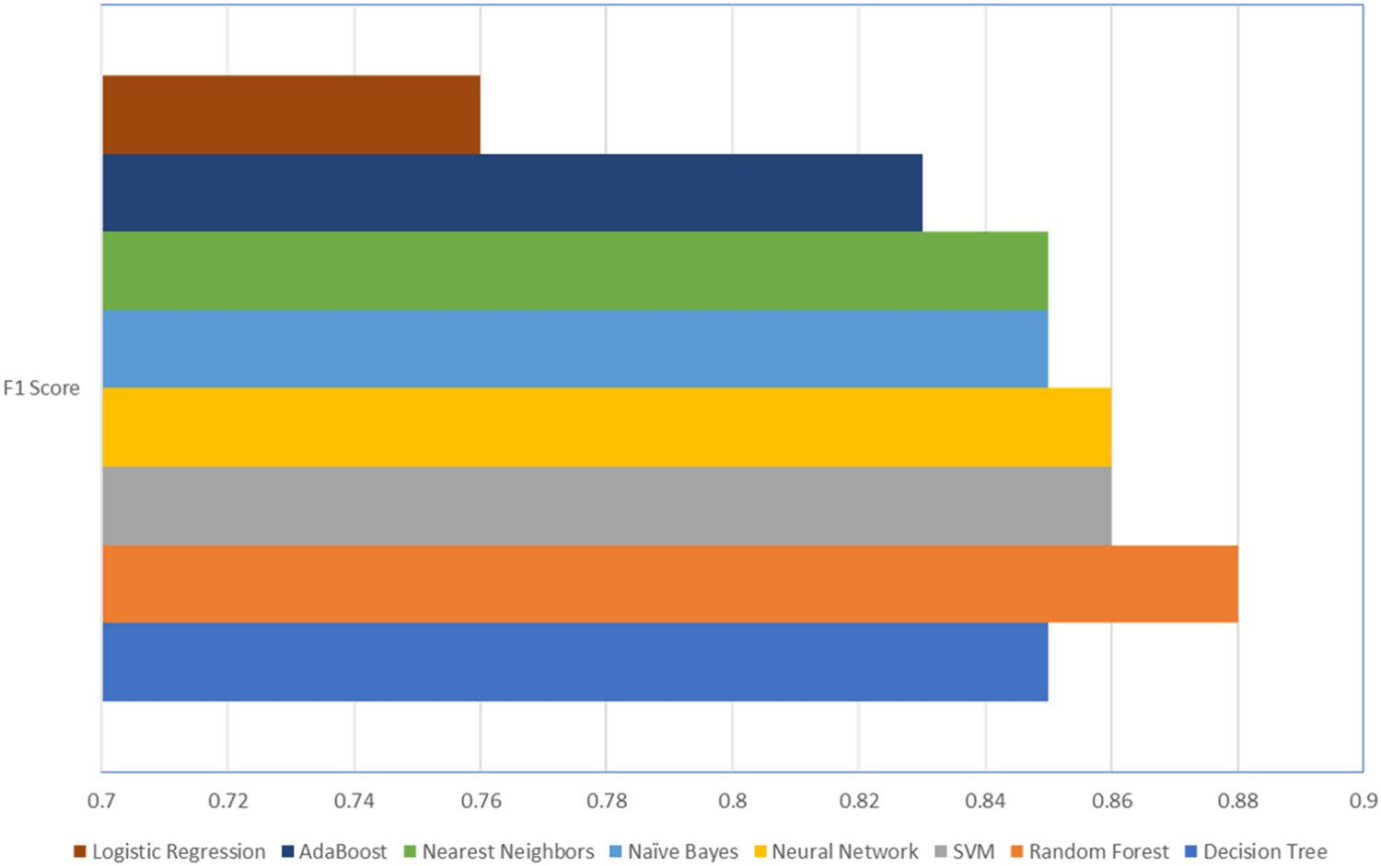
F1 Score provided by the eight algorithms considered in the analysis.

As stated earlier, we have considered eight common classification algorithms, and the results of our experiments are summarized in Table 3 and also shown in a graph, Fig. 11. As seen in the table, the Decision Tree and Random Forest algorithms provide the highest accuracy of 93%, compared to the others, followed by the SVM and Neural Networks (with one hidden layer) with 92% accuracy. In comparison, the Logistic Regression algorithm shows the lowest accuracy of 87%.

**Table 3:** Performance evaluation of each algorithm.

Algorithm	Accuracy (%)	Precision	Recall	F1 score
Decision tree	93	0.88	0.88	0.85
Random forest	93	0.88	0.88	0.88
SVM	92	0.82	0.90	0.86
Neural network	92	0.85	0.90	0.86
Naïve Bayes	91	0.85	0.85	0.85
Nearest neighbors	90	0.91	0.79	0.85
AdaBoost	90	0.79	0.87	0.83
Logistic regression	87	0.67	0.88	0.76

The confusion matrix is another way to evaluate our results, and the confusion matrix result of each of the eight classification algorithms are shown in Table 4. With regards to the precision, Recall, and F1 Score, F1 Score is considered a very well evaluation metric to observe the balance between precision and recall, especially when there is an imbalanced class distribution with a large number of actual False observations, as shown in Fig. 9. From the results in Table 3, we can see that the Random Forest algorithm has the highest F1 Score of 0.88, followed by SVM and Neural Network with a 0.866 F1 score.

**Table 4:**
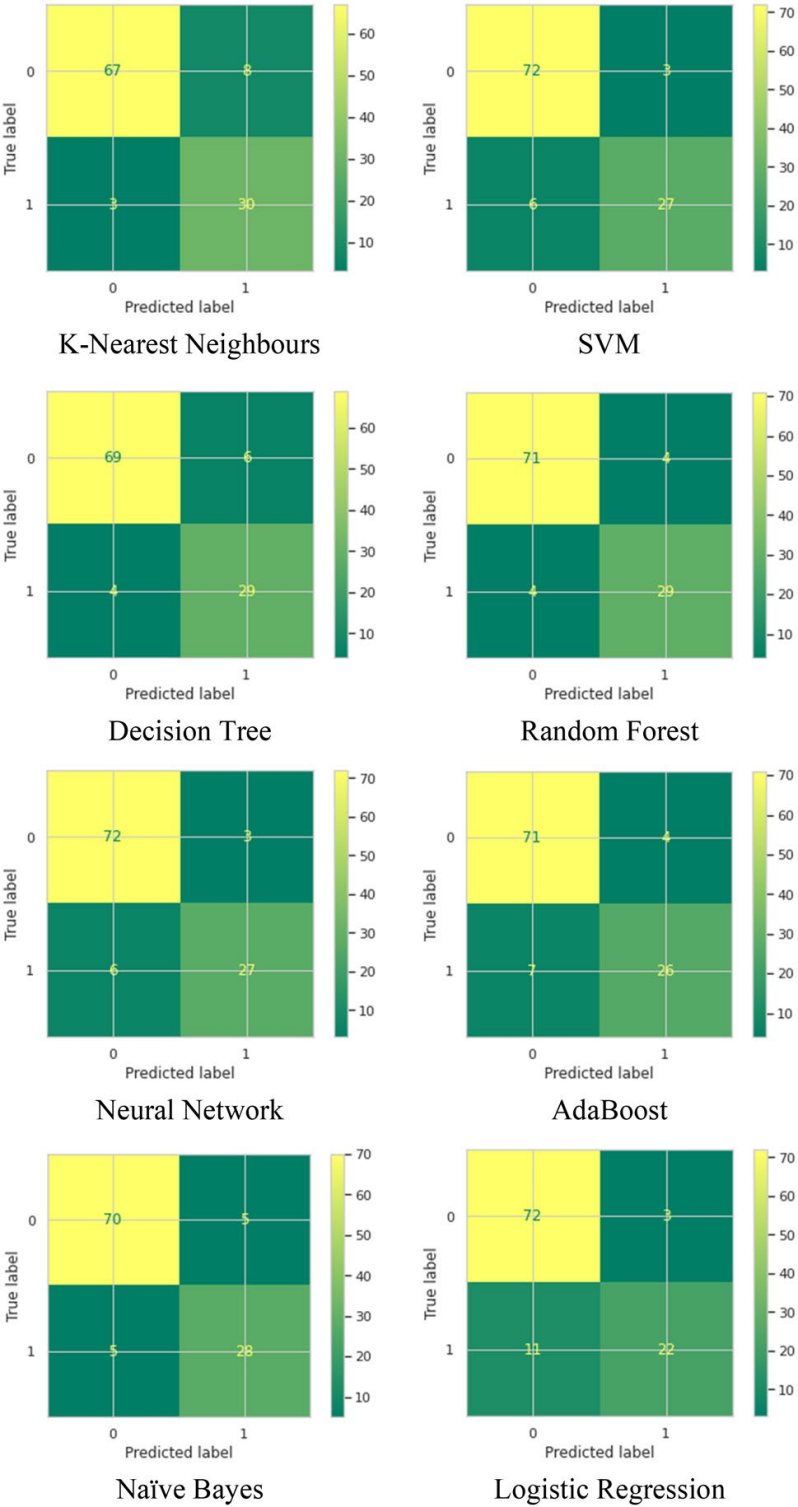
Confusion matrix of the eight classification algorithms considered in the study.

**Figure 12:**
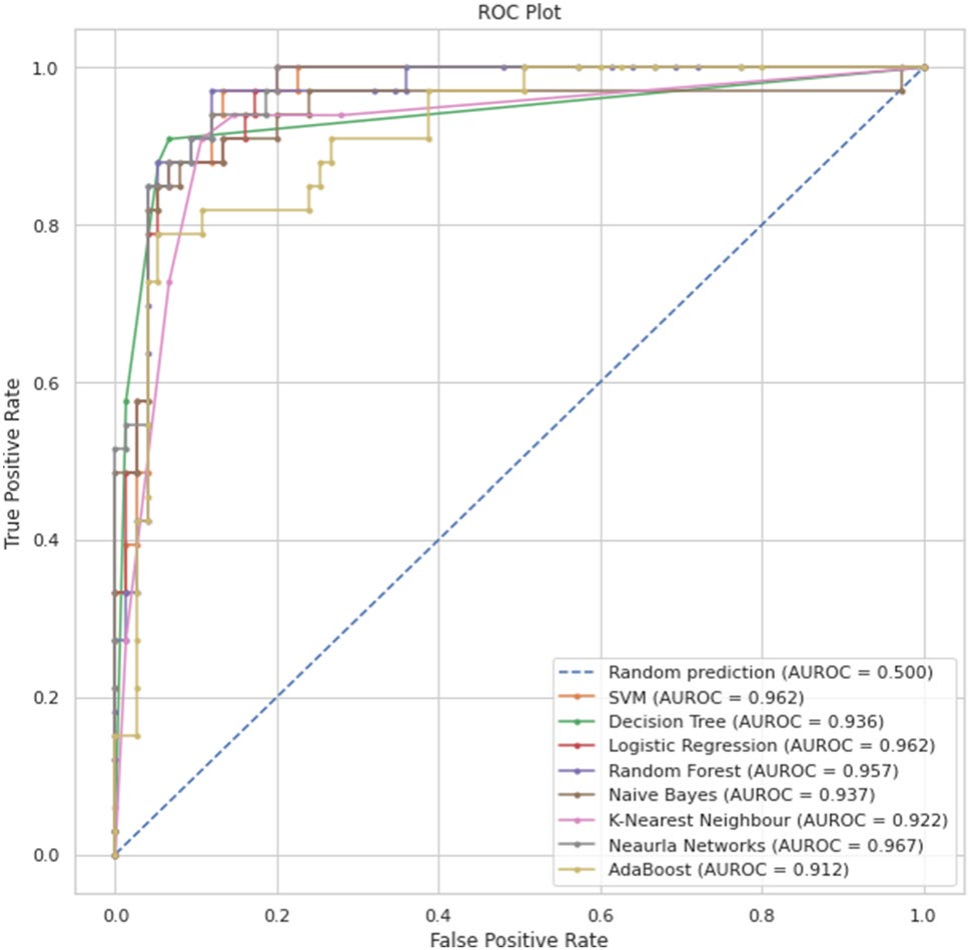
AUC of the eight classifier models.

For accurate performance comparison of the eight classifier models considered in our study, and bearing in mind the imbalanced dataset used here, we have also calculated the Area Under the Curve (AUC) as it is an important metric and indicator of the models' performance. For that, AUC was derived for each of the eight classifier models, AUC for each model, and all in one graph for comparison purposes in Fig. 12. From the results displayed in that figure, it can be seen that the SVM, Neural Network, and Random Forest provide the best prediction results.

It is worth mentioning that we have also investigated the case when only features with the highest correlation (those with 25% and above) have been kept and all other remaining features have been dropped, to end up with the remaining ones: Elongation, altitudeDifference, MoonAltitude, Sun_Moon_Lag, atmosphere. It has been noticed a similar trend as the results shown earlier, i.e., SVM, Neural Network along with K-Nearest Neighbor and Naïve Bayes providing the highest accuracy (note that the two latter models have improved their accuracy when high correlation has been taken into account).

Referring to the results obtained in^44^ and^45^, which were the only studies that used some machine learning algorithms in the prediction of the new crescent Moon, the comparison of the results is irrational. The dataset in^45^ was restricted to only one country and employed only one ML algorithm. However, the study in^44^ used dataset different from ours; our dataset is more accurate and complete, as mentioned earlier. Both datasets are imbalanced. Therefore, the accuracy was not the only performance metric that was considered but we took other important performance measures like Recall, F1 Score, AUC. Our results show that Recall, for instance, is higher than that found in^44^. Moreover, in our study we considered more machine learning algorithms to seek better model.

## Conclusion and future work

Deciding on the first day of Ramadan month has always been a challenge for Muslims across the world, resulting in a division of Muslims fasting on different dates. Usually, the start of Ramadan is marked by the sighting of the new crescent Moon using different technologies and methods. This paper has investigated the use of machine learning classification algorithms to predict the visibility of the new crescent Moon based on the observations of our dataset built from the Islamic Crescents Observation Project observations with extra information and observations added. The present study has shown that the three models, SVM, Neural Network, and Random Forest provide the best prediction results with high accuracy exceeding 91%.

As future work, we plan to enrich our dataset with more balanced records to enhance the prediction results. Also, we will include other classification algorithms for further investigation.

## Data Availability

The datasets used and/or analysed during the current study available from the corresponding author on reasonable request.

## Code availability

The code will be available from the corresponding author on reasonable request.
